# Predicting orthophosphate in feces and manure from dairy cattle

**DOI:** 10.3168/jdsc.2023-0388

**Published:** 2023-09-22

**Authors:** Joyce L. Marumo, P. Andrew LaPierre, Andres F. Ortega, Michael E. Van Amburgh

**Affiliations:** Department of Animal Science, Cornell University, Ithaca, NY 14853

## Abstract

Dairy cattle excreta are a valuable source of orthophosphate (Ortho-P), an inorganic form of phosphorus (P) that is readily available for microorganisms, plant growth, and development. There is, however, a growing environmental concern about the potential negative environmental impact of excessive amounts of Ortho-P excretion, which can lead to the eutrophication of water bodies. As a result, the development of mathematical equations to quantify and manage Ortho-P excretion on dairy farms could prove valuable for environmental sustainability. This study aimed to use literature data to develop empirical predictions for Ortho-P (g/kg dry matter [DM]) excretion using total P (TP [g/kg DM]) content of dairy cattle feces (Ortho-P_f_) and manure (Ortho-P_m_). Data sets from studies that evaluated and characterized the different forms of P in feces and manure from dairy cattle were compiled. After outlier exclusion, the final retained database for feces included 37 treatment means from 4 published papers while the manure comprised 23 treatment means from 7 published papers. A linear-mixed model was used to develop the predictive equations, incorporating the random effect of the study. A leave-one-out cross-validation procedure was used to evaluate the predictive ability of the developed models, whereby studies were regarded as folds. The fecal equation was determined as Ortho-P_f_ (g/kg DM) = −2.447 (0.572) + 0.966 (0.083) × TP (g/kg DM) (R^2^ = 0.79) and resulted in a root mean square prediction error as a percentage of mean observed value (RMSPE, %) of 32.8% and error due to random sources of 97.6%. Additionally, the manure equation was determined as Ortho-P_m_ (g/kg) = −0.204 (0.446) + 0.590 (0.065) × TP (g/kg) (R^2^ = 0.77) and had an RMSPE of 43.3% with a random error of 93.9%. Both models revealed minimal mean and slope biases on feces and manure data. Findings suggest that these sets of equations can be used to estimate excreted Ortho-P from total excreted P, helping nutritionists and farmers to understand the impact of dietary P changes on the environment. Further, these equations can be incorporated into extant models such as the Cornell Net Carbohydrate and Protein System (CNCPS) to aid in understanding and mitigating P and Ortho-P excretion from dairy cattle and to clarify the portion of P that migrates more rapidly into watersheds.

## Highlights

• Overall, TP and Ortho-P feces concentrations are higher than that of manure in dairy cattle.
• Simple linear mixed models to predict fecal and manure Ortho-P using TP in dairy cattle were developed.
• Both proposed Ortho-P models for feces and manure showed the absence of systematic bias (mean and slope biases).

## Graphical Abstract

**Figure:**
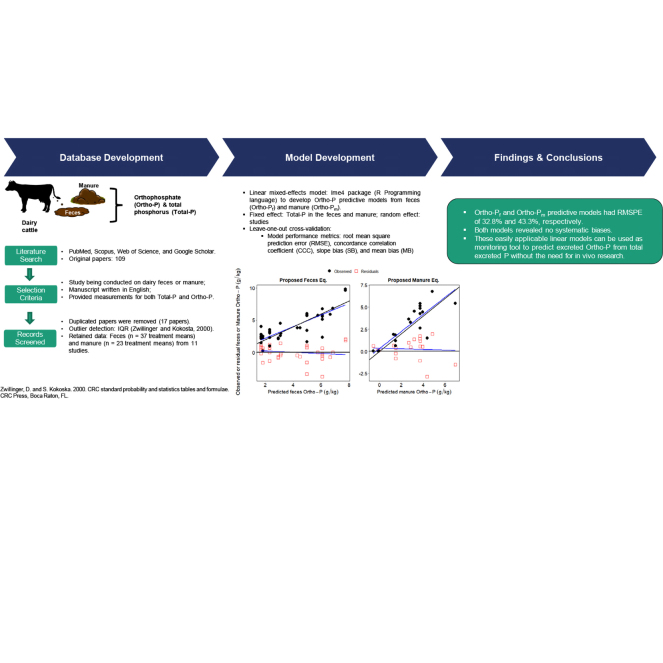
**Summary:** Orthophosphate (Ortho-P) is an essential nutrient for plant growth and development when applied in appropriate amounts; however, it can be detrimental to the environment by causing eutrophication of waterbodies when present in excess. Empirical mathematical equations can be used to manage and monitor its use and discharge to eliminate negative impact on the environment. To address this issue, predictive models of Ortho-P (g/kg dry matter) release using total phosphorus (TP; g/kg) content of dairy cattle feces (Ortho-Pf) and manure (Ortho-Pm) were constructed. Both proposed models showed minimal systematic bias. Therefore, these easily applicable models can be used without the need for costly, laborious laboratory analysis.

Dietary phosphorus (P) is an essential macro-mineral in dairy cattle for diverse physiological processes including but not limited to energy transfer via ATP, bone mineralization, lipid metabolism (Berndt and Kumar, 2009), reproduction (Wang et al., 2014), and digestion of cellulose by ruminal microorganisms (Burroughs et al., 1951). The National Research Council (NRC, 2001) recommends a range between 0.32% and 0.42% (DM basis) of P feeding level for dairy cattle depending on the animal's production level (NASEM, 2021). Despite these guidelines, farmers and nutritionists often overfeed inorganic P supplements to enhance P availability to meet cattle requirements for production and reproduction (Dou et al., 2003; Bradford et al., 2009), resulting in excess P excreted in the feces (Wang et al., 2014) and manure (Bradford et al., 2009) as well as increased feed costs and environmental concerns. Orthophosphate (**Ortho-P**) is an inorganic form of P that is soluble in water, making it readily available to animals, plants, and microorganisms for growth and development (Plaxton and Tran, 2011; Victor Roch et al., 2019). However, the effectiveness of P absorption in animal diets is influenced by the conversion/transformation of feed P into inorganic P or more digestible forms of organic P (Toor et al., 2005). There is growing concern regarding excessive supplementation of P in diets and fertilizer applications due to the adverse impact on the environment (Liu et al., 2017). As such, overuse of P could lead to an increased loss of Ortho-P in feces and manure that might cause eutrophication of surface waters (Kent et al., 2020), which can be harmful to aquatic ecosystems by promoting the growth of unwanted plants and algae (Knockaert, 2022). Manure-P, fecal-P, and Ortho-P can be quantified using laboratory techniques such as colorimetric assays and spectrometry, which can be laborious, costly, and time consuming (Wilschefski and Baxter, 2019; Wieczorek et al., 2022). Quantification of TP output in feces and manure by dairy cattle has been proven to be challenging; therefore, previous research has put effort into developing mathematical models for these excreta (Klop et al., 2013; Alvarez-Fuentes et al., 2016). Reliably predicting the excretion of TP as Ortho-P from feces and manure would aid in evaluating the effectiveness of P utilization in animal diets. To our knowledge, only the Toor et al. (2005) study developed predictive equations for Ortho-P concentration based on TP in excreta, but these equations have not been validated and were primarily derived from laboratory experiments. Evaluating predictive equations is crucial for accurately estimating the amount of fecal and manure Ortho-P released from TP in dairy cattle. To address this, the development of simple and practical mathematical models can be helpful to manage and monitor the potential environmental effect of P and Ortho-P excretion from dairy cattle. These models could be integrated into nutrition models such as Cornell Net Carbohydrate and Protein System (**CNCPS**; Van Amburgh et al., 2015), the National Academies of Sciences, Engineering, and Medicine (NASEM, 2021), and other nutrient prediction models to provide dairy farmers and nutritionists information on current Ortho-P levels and how dietary modifications can affect excretion of these nutrients. Also, the CNCPS has predictions for P excretion in urine, feces, and manure (Fox et al., 2004) and this study would extend and refine those predictions to provide more information to nutritionists, dairy producers, and crop planners. Properly used, the models can provide P and Ortho-P intake and excretion inventories that could be useful for crop and nutrient management planners to understand how much P needs to be allocated to the available land base. Therefore, the objective was to develop and evaluate empirical mathematical models that predict Ortho-P excretion using modeled total P (**TP** [g/kg]) content in dairy cattle feces (**Ortho-P_f_**) and manure (**Ortho-P_m_**).

A literature search was conducted in Web of Science (https://www.webofscience.com), Scopus (https://www.scopus.com), Google Scholar (https://scholar.google.com), and PubMed (https://pubmed.ncbi.nlm.nih.gov), using the combination of the 7 search terms: “orthophosphate,” “ortho-phosphate,” “phosphorus,” “phosphate,” “manure,” “feces,” “faeces,” “cattle,” and “cow.” These searches resulted in a total of 109 papers from 2001 to 2023.

Studies were included if (1) work was conducted on dairy manure, feces, or both; (2) the manuscript was written in English; (3) they provided measurements of both TP and Ortho-P in either unit of measure (mg/kg or g/kg). All expressed Ortho-P and TP units were converted to grams per kilogram. Studies on beef cattle were excluded from the final data analysis (3 papers). Duplicates and null entries (17 papers) were excluded using Endnote 20 (version 20.4.1) resulting in a total of 92 papers being retained in the data for further screening. The remaining papers were further screened by reading abstracts, full text, and results. The final data set for the feces and manure comprised 11 papers, with 1 paper that reported Ortho-P and TP measurements in both manure and feces (Toor et al., 2005). In the context of this paper, feces specifically pertain to the excreted material from dairy cows, containing various nutrients including P and Ortho-P. Manure, on the other hand, encompasses a broader concept and refers to the mixture of feces along with other organic materials such as urine, bedding materials, and microbial activity byproducts from the barn. The assessment of outliers in the database was conducted using the interquartile range method (Zwillinger and Kokoska, 2000) with a factor of 1.5 to be considered an extreme value. One study was identified as an outlier and removed from the final analysis.

Data analysis was conducted using the R programming language (R Core Team, 2022; R version 4.2.1 [2022–06–23 ucrt]) in Rstudio environment (RStudio Team, 2022). Simple linear-mixed models were fitted using the ‘lmer function' in the lme4 package, with the study as the random effect and TP (g/kg) in the feces and manure as the fixed effect based on the equation

[1] *Y* = *β*_0_ + *β*_1_*X*_1_ + *S_i_* + *e_ij_*,

where *Y* is the dependent variable of Ortho-P (g/kg), *β*_0_ is the fixed effect of the random intercept, *X*_1_ is the fixed effects of the independent variable (TP; g/kg) and *β*_1_ is the corresponding slope, *S_i_* is the random effect of the studies, and *e_ij_* is the random error.

Leave-one-out cross-validation (**LOOCV**) was performed to evaluate the prediction accuracy of the developed models with the study considered as the folds, whereby 1 study was used as the validation data and the remaining studies were used as the training data in each iteration (Ribeiro et al., 2020). The performance metrics of the models were calculated using the predictions generated from LOOCV (Table 1). The combination of the metrics determined included Lin's concordance correlation coefficient (**CCC**; Lin, 1989) calculated with the epiR package (Stevenson et al., 2022), total mean square prediction (**MSPE**, Equation 2; Bibby and Toutenburg, 1977), and root mean squared prediction error (**RMSPE**) expressed as a percentage of the observed mean (g/kg). The RMSPE % measures the agreement between the observed and predicted values. To detect the systematic biases in the predictions, the total MSPE was then decomposed into mean bias (**MB**, Equation 3) and slope bias (**SB**, Equation 4) expressed as the percentage of the MSPE and error due to random sources (**ED**, Equation 5). The smaller RMSPE value indicates better overall model predictive accuracy, whereas a higher CCC value indicates a successful model performance.

[2] $$MSPE=\frac{\sum_{i=1}^{n}{\left(y_{i}-{\hat{y}}_{i}\right)}^{2}}{n},$$

where *y_i_* is the observed value of the Ortho-P variable for the *i*th observation, and ${\hat{y}}_{i}$ is the predicted value of the Ortho-P variable for the *i*th observation.

[3] $$MB={\left(\bar{P}-\bar{O}\right)}^{2},$$

[4] $$SB={\left(Sp-r\times So\right)}^{2},$$

[5] $$ED=\left(1-r^{2}\right)\times So^{2},$$

where $\bar{P}$ and $\bar{O}$ are the predicted and observed means, respectively; *Sp* and *So* are predicted and observed standard deviations, respectively, and r is the Pearson correlation coefficient.

**Table 1:** Orthophosphate (g/kg) proposed prediction models and performance evaluations using feces (n = 37) and manure (n = 23) databases^1^

Proposed model^2^	Predictive equation^3^	Model performance^4^					
		RMSPE, %	MB, %	SB, %	ED, %	CCC	R^2^_adjusted_
Feces (Ortho-P_f_)	−2.447 (0.572) + 0.966 (0.083) × TP (g/kg)	32.8	0.05	2.31	97.6	0.81	0.79
Manure (Ortho-P_m_)	−0.204 (0.446) + 0.590 (0.065) × TP (g/kg)	43.3	5.68	0.41	93.9	0.87	0.77

1 Studies used to generate the feces and manure databases: Hansen et al., 2004; Toor et al., 2005; McDowell and Stewart, 2005a,b; Pan et al., 2006; He et al., 2007; McDowell et al., 2008; Qureshi et al., 2008; He et al., 2009; Liu et al., 2014; Cade-Menun et al., 2015.

2 Ortho-P_f_ = fecal orthophosphate model; Ortho-P_m_ = manure orthophosphate model.

3 TP = total phosphorus concentration (g/kg).

4 RMSPE = root mean square prediction error, expressed as the percentage of the observed mean orthophosphate (g/kg); MB = mean bias, expressed as the percentage of the total mean square prediction error; SB = slope bias, expressed as the percentage of the total mean square prediction error; ED = error due to random sources as the percentage of the total mean square prediction error; CCC = concordance correlation coefficient. Figure 1 demonstrates the performance of the proposed fecal and manure orthophosphate models.

In feces, TP excreted ranged from 4.2 to 11.3 g/kg with a mean of 6.6 ± 2.02 g/kg, whereas Ortho-P ranged from 0.2 to 9.8 g/kg with an average of 3.9 ± 2.19 g/kg. On the other hand, the TP excreted in manure ranged from 0.05 to 11.0 g/kg with a mean of 4.3 ± 3.36 g/kg, whereas Ortho-P released ranged from 0.02 to 6.8 g/kg with a mean of 2.5 ± 2.24 g/kg. Overall, fecal TP and Ortho-P concentrations were greater than that in manure, but variability was slightly greater in manure. The difference between fecal and manure Ortho-P may be explained by fecal-P diluted by manure foreign materials. Manure composition is highly variable as it is composed of different low-P foreign sources such as urine, bedding, and water (Toor et al., 2005) and is influenced by several factors such as animal diet, breed, and compositing phase (Barnett, 1994a). These factors could explain the greater variability and lower TP in manure, but they cannot be confirmed in the present study as they were not reported in the literature. Our findings are consistent with that of Toor et al. (2005) who reported higher P in feces than in manure with a range of 5.7–9.5 g/kg and 2.5–8.9 g/kg, respectively. In contrast to our findings, Sharpley and Moyer (2000) reported a lower manure TP of 3.5 ± 2.01 g/kg, averaging 24 samples collected over the 2-yr period from the same farm. This could be explained by differences in diet characteristics, extraction methods, and sampling methods of feces and manure (Cade-Menun, 2011), which were not often provided in the current study.

Orthophosphate (Ortho-P_f_ and Ortho-P_m_) model prediction equations and performance metrics for feces and manure are in Table 1. In agreement with previous findings (Powell et al., 2001; Toor et al., 2005), our results revealed a positive relationship between TP and Ortho-P in both feces (Ortho-P_f_: *r* = 0.89, *P* < 0.001) and manure (Ortho-P_m_: *r* = 0.91, *P* < 0.001). These significant associations could partly be attributed to the fact that an increase in dietary P has been shown to increase the total concentration of fecal-P (Alvarez-Fuentes et al., 2016), but also, more notably, increases the proportion of water-soluble P (Dou et al., 2002, 2003), which is particularly susceptible to environmental loss through leaching and surface runoff. However, the findings regarding excreted water-soluble P in feces are inconsistent. For instance, Dou et al. (2003) examined fecal and dietary samples from 75 commercial dairy farms in the United States and showed that a 1-unit incremental increase in dietary P (g P/kg of feed DM) led to an increase of 1.89 g P/kg of fecal DM, with 1.00 g out of 1.89 (53%) being from the water-soluble fraction. On the other hand, Dou et al. (2002) reported the majority (>80%) of excess P excreted in feces is water soluble, and these discrepancies might be attributable to differences in laboratory extraction methods. Ensuring that the cow's diet contains the appropriate amount of P, as per its requirements for physiological status (e.g., lactation, pregnancy, growth), can lead to significant reductions in excretion and potential environmental risks (Morse et al., 1992; Kebreab et al., 2013). As per the NRC (2001) guidelines, the recommended maximum dietary P content for cows during the first few weeks of lactation is 0.42%.

Our Ortho-P_f_ model indicates that every 1 g/kg of fecal TP excretion would result in a 0.97 ± 0.08 g/kg (0.10%) increase in Ortho-P release in dairy cattle (Table 1), and these findings are comparable to that of Toor et al. (2005) who observed a slope of 0.99 g/kg from fecal samples collected from 6 commercial dairy farms in the United States. In addition, a similar observation was identified in the present study for the manure whereby concentrations of TP increased with increasing manure Ortho-P concentration with a slope of 0.59 g of Ortho-P/kg. Both Ortho-P_f_ and Ortho-P_m_ models revealed no systematic biases (Table 1 and Figure 1), but the Ortho-P_f_ model had lower RMSPE (32.8%) and higher random source of error (ED = 97.6%) than the Ortho-P_m_ model (RMSPE = 43.3% and ED = 93.9%; Table 1). These results suggest that lowering fecal TP will lower the Ortho-P in both feces and manure.

**Figure 1:**
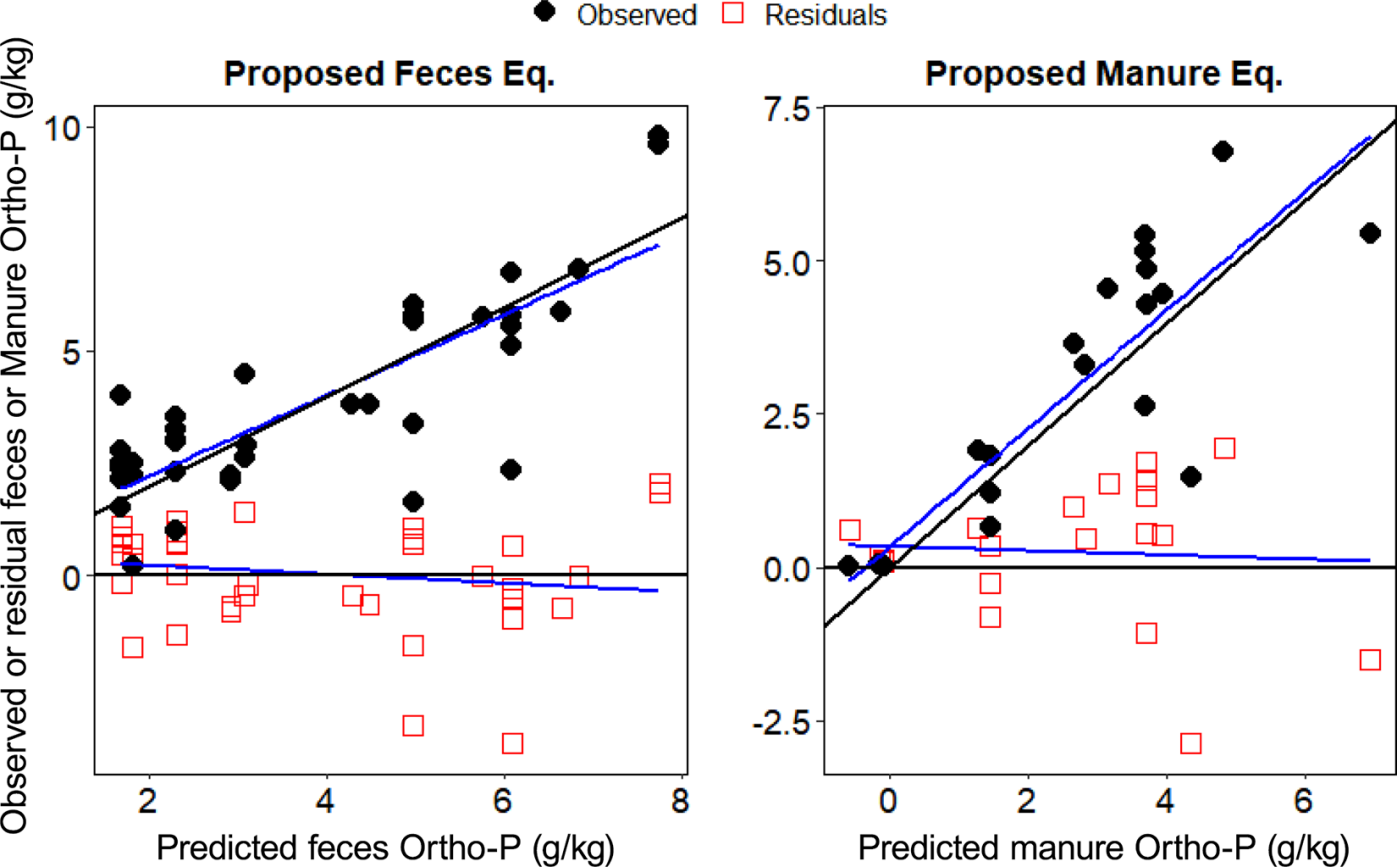
Plots of observed versus predicted fecal or manure orthophosphate (Ortho-P; g/kg; black diamonds), and residuals (red squares: observed − predicted values) versus predicted Ortho-P (g/kg; black diamond shapes represent the observed fecal or manure orthophosphate) generated from fecal (n = 37) and manure databases (n = 23). The solid blue lines indicate the relationship between predicted and observed fecal or manure Ortho-P and predicted values and the residuals. The solid black lines represent the line whereby predicted = observed values.

In the present study, fecal and manure TP concentrations accounted for 79% and 77% of variations in Ortho-P_f_ and Ortho-P_m_, respectively (Table 1). However, the magnitude of the R^2^ for both Ortho-P_f_ and Ortho-P_m_ models in our study is lower than that reported by Toor et al. (2005) of 96% and 91%, respectively. Due to interstudy variability in the current study, these results are not surprising because their study was conducted under laboratory conditions. In laboratory experiments, researchers can control for multiple factors that may affect the outcome of their research, which is challenging in studies that involve little or no intervention like the current study.

The higher coefficient of determination and lower RMSPE in the Ortho-P_f_ model than Ortho-P_m_ model postulates that TP is a better predictor of variation in Ortho-P concentration in feces. The Ortho-P_f_ model may have shown better prediction than Ortho-P_m_ because feces provide a direct measure of the animal's excretion as it is composed of undigested feed and other solid materials that are not affected by other foreign sources such as urine, bedding, and so on. In addition, fecal Ortho-P likely reflects changes in dietary P composition and the animal's P status (McDowell et al., 2008; Dou et al., 2010). This agrees with Dou et al. (2002), who reported that readily soluble P (inorganic P) can be a reliable reflection of dietary P consumption, bioavailability, and utilization. In addition to recommended periodic feed analysis, our proposed models might offer benefits by providing additional information on the P availability to help manage P plans of commercial dairy farms to reduce excess P excretion in dairy cattle. The Ortho-P_f_ model can be used to assess the impact of the diet on individual animals, while the Ortho-P_m_ model can evaluate the P efficiency of the overall feeding program for the dairy farm or Ortho-P levels in the effluent/manure lagoon.

The present study developed simple regression models that can predict excreted Ortho-P from total excreted P in feces and manure in dairy cattle. Both models can be used to predict Ortho-P excretion depending on the specific context and goals or management practices. When used in conjunction with TP excretion models, these models can serve as a nutrient management and monitoring tool on a dairy farm and could be incorporated into decision-support tools such as nutrition models to enable precision ration formulation. By doing so, excess dietary P can be assessed, and fecal and manure Ortho-P can be reduced or understood without compromising animal productivity. This approach would help farmers save money while also reducing the risk to the environment. Moreover, these models can be used without conducting costly and laborious experiments and have shown no systematic error.
